# HER inhibitor promotes BRAF/MEK inhibitor-induced redifferentiation in papillary thyroid cancer harboring BRAFV600E

**DOI:** 10.18632/oncotarget.15773

**Published:** 2017-02-28

**Authors:** Lingxiao Cheng, Yuchen Jin, Min Liu, Maomei Ruan, Libo Chen

**Affiliations:** ^1^ Department of Nuclear Medicine, Shanghai Jiao Tong University Affiliated Sixth People’s Hospital, Shanghai 200233, China; ^2^ Department of Nuclear Medicine, Shanghai Chest Hospital, Shanghai Jiao Tong University, Shanghai 200030, China

**Keywords:** papillary thyroid cancer, redifferentiation, iodine, glucose, dabrafenib

## Abstract

Redifferentiation therapy with BRAF/MEK inhibitors to facilitate treatment with radioiodine represents a good choice for radioiodine-refractory differentiated thyroid carcinoma, but recent initial clinical outcomes were modest. MAPK rebound caused by BRAF/MEK inhibitors-induced activation of HER2/HER3 is a resistance mechanism, and combination with HER inhibitor to prevent MAPK rebound may sensitize BRAF^V600E^-mutant thyroid cancer cells to redifferentiation therapy. To evaluate if inhibiting both BRAF/MEK and HER can produce stronger redifferetiation effect, we tested the effects of BRAF/MEK inhibitor dabrafenib/selumetinib alone or in combination with HER inhibitor lapatinib on the expression and function of iodine- and glucose-handling genes in BRAF^V600E^-positive BCPAP and K1 cells, using BHP 2-7 cells harboring RET/PTC1 rearrangement as control. Herein, we showed that lapatinib prevented MAPK rebound and sensitized BRAFV600E-positive papillary thyroid cancer cells to BRAF/MEK inhibitors. Dabrafenib/selumetinib alone increased iodine-uptake and toxicity and suppressed glucose-metablism in BRAF^V600E^-positive papillary thyroid cancer cells. When lapatinib was added, more significant effects on iodine- and glucose-handling gene expression, cell membrane location of sodium/iodine symporter as well as radioiodine uptake and toxicity were observed. Thus, combined therapy using HER inhibitor and BRAF/MEK inhibitor presented more significant redifferentiation effect on papillary thyroid cancer cells harboring BRAF^V600E^ than BRAF/MEK inhibitor alone. *In vivo* and clinical studies assessing such combined targeted redifferentiation strategy were warranted.

## INTRODUCTION

Thyroid cancer is the most common endocrine malignancy with an increasing incidence over the past few decades, with differentiated thyroid carcinoma (DTC) occurring in more than 90% of cases [1]. For most cases of DTC, disease courses are indolent and cures are usually achieved with standard therapy including surgery, radioiodine (^131^I) treatment and TSH suppression. Unfortunately, in nearly 5% of DTC patients, tumors go through a dedifferentiation process, accompanied by the silencing of iodine handling genes such as sodium/iodine symporter (NIS), TSH receptor (TSHR), thyroperoxidase (TPO), and thyroglobulin (Tg), which ultimately causes reducing of ^131^I uptake and failure of ^131^I treatment [2, 3]. These radioiodine-refractory differentiated thyroid carcinoma (RR-DTC) are usually aggressive with unresectable metastatic lesions, which becomes the main cause of thyroid cancer-related morbidity and mortality [2].

Patients with RR-DTC usually show positive FDG-avid lesions on ^18^FDG-PET/CT due to intense glucose metabolism [4], which is partially due to the enhanced expression level of GLUT1 gene in less differentiated thyroid carcinomas [3, 5]. Decreases of GLUT1 expression have been previously detected by our group when reinducing thyroid cancer iodine-uptake with multikinase inhibitors, sorafenib/cabozantinib, which also target the MAPK pathway [6].

Radioiodine-refractory and iodine-handling gene silencing in thyroid cancer is associated with aberrant activating of MAPK pathway, which is frequently caused by the *BRAF*^V600E^ mutation [4]. This has already been overcome on levels of cell lines and animal models by treatment with various kinds of BRAF and MEK inhibitors [6–8]. Furthermore, in clinical trials, blockade of MAPK pathway with the MEK inhibitor, selumetinib, and BRAF inhibitor, dabrafenib, has been demonstrated to cause redifferentiation and enhancement of radioiodine uptake in RR-DTC [9, 10]. On the basis of previous results, two randomized, placebo-controlled trials have been initiated to determine whether selumetinib followed by ^131^I therapy prevents disease recurrence following initial surgery in patients with high-risk DTC (NCT01843062), and/or enhances tumor regression in patients with ^131^I-avid metastatic DTC (NCT02393690) [11]. However, existing results showed that only part of patients with *BRAF*^V600E^-mutant disease developed new uptake with MEK or BRAF inhibition, and even fewer had an increased uptake that exceeded the dosimetry threshold for radioiodine treatment [9, 10].

Why MEK/BRAF inhibitors failed to meet high expectation in clinical trials of redifferentiation is not clear. Feedback mechanism that limits the effects of RAF/MEK inhibitors on downstream signaling in thyroid cancer may be a proper explanation for drug resistance in such redifferentiation therapy. Recent studies implicate that feedback-reactivated HER signaling causes activation of the PI3K/AKT, MEK/MAPK, and JAK/STAT signaling pathways as well as Src kinase, which ultimately causes treatment failure [12–14]. As is demonstrated in Fagin’s study, thyroid tumor cells harboring the *BRAF*^V600E^ over express HER signaling pathway when treated with BRAF/MEK inhibitors, reactivating ERK and/or AKT [15]. Combination of HER inhibitor to BRAF/MEK inhibitor solves the problem of drug resistance [15]. Therefore, we hypothesize that greater redifferetiation effect may be achieved via dual inhibition of BRAF/MEK and HER than sole inhibition of BRAF/MEK.

In the present study, we investigate whether simultaneous inhibition of BRAF/MEK with dabrafenib/selumetinib and HER with lapatinib has stronger redifferetiation effect and lead to greater augment in radioiodine uptake than sole inhibition of BRAF/MEK. Meanwhile, we observe whether GLUT expression and FDG uptake can be suppressed to a lower extent after simultaneous inhibition, in order to judge the potential of ^18^F-FDG PET in revealing efficacy of redifferentiation therapy.

## RESULTS

### Gene sequencing

Mutant *BRAF*^V600E^ and wild-type *NRAS* genes were confirmed in BCPAP cells. Mutant *BRAF*^V600E^, *PIK3CA* and wild-type *NRAS* gene were confirmed in K1 cells. RET/PTC1 rearrangement with wild-type genes of *BRAF* and *NRAS* were confirmed in BHP 2-7 cells. Genetic alterations of these cell lines are presented in Supplementary Figure 1.

### Effects on cell proliferation and cell cycle

As is shown in Supplementary Figure 2, the half maximal inhibitory concentration (IC_50_) of dabrafenib in BCPAP cells, K1 cells and BHP 2-7 cells were 232 nM, 146 nM, 315 nM, respectively. And the IC_50_ of selumetinib in BCPAP cells, K1 cells and BHP 2-7 cells were 9274 nM, 16270 nM, 23370 nM, respectively. IC_50_ of lapatinib in the three cell lines were 9134 nM, 11330 nM and 4250 nM, respectively. Lapatinib markedly sensitized the three cell lines to dose-dependent inhibition by the BRAF/MEK inhibitor. When 1μM lapatinib was added to BCPAP cells, K1 cells and BHP 2-7 cells, the IC_50_ of dabrafenib decreased significantly to 74 nM, 47 nM and 201 nM, respectively, and the IC_50_ of selumetinib dropped significantly to 2395 nM, 1320 nM and 8563 nM, respectively.

We had set a Concentration gradients in pre-experiments were set and dabrafenib at 0.1 μM, selumetinib at 2.5 μM and lapatinib at 1 μM were found to induced preferable redifferentiation effect in BCPAP and K1 cells. Such concentrations were used in the following experiments.

When treated with DMSO, 46% of the BCPAP cells were found to be in the G1 phase, 38.7% in the S phase, and 14.9% in the G2 phase; 67.5% of the K1 cells were found to be in the G1 phase, 27.9% in the S phase, and 5.6% in the G2 phase; 55.0% of the BHP 2-7 cells were found to be in the G1 phase, 30.7% in the S phase, and 14.3% in the G2 phase. BCPAP cells and K1 cells treated with 0.1 μM dabrafenib alone or in combination with 1 μM lapatinib for 24 h significantly differ in G1/S phase content compared with the DMSO control (*P* < 0.01) (Supplementary Figure 3). When treated with 2.5 μM selumetinib alone or in combination with 1 μM lapatinib, BCPAP cells and K1 cells were arrested in the G1 phase with statistical significance (*P* < 0.01) compared with the amount of cells in the G1/S phase in the DMSO control (Supplementary Figure 3). Neither BRAF/MEK inhibition nor dual inhibition of BRAF/MEK and HER induced marked cell cycle arrest in the G1 phase in BHP 2-7 cells (Supplementary Figure 3).

### Prevention of MAPK rebound induced by BRAF/MEK inhibitor

**Figure 1:**
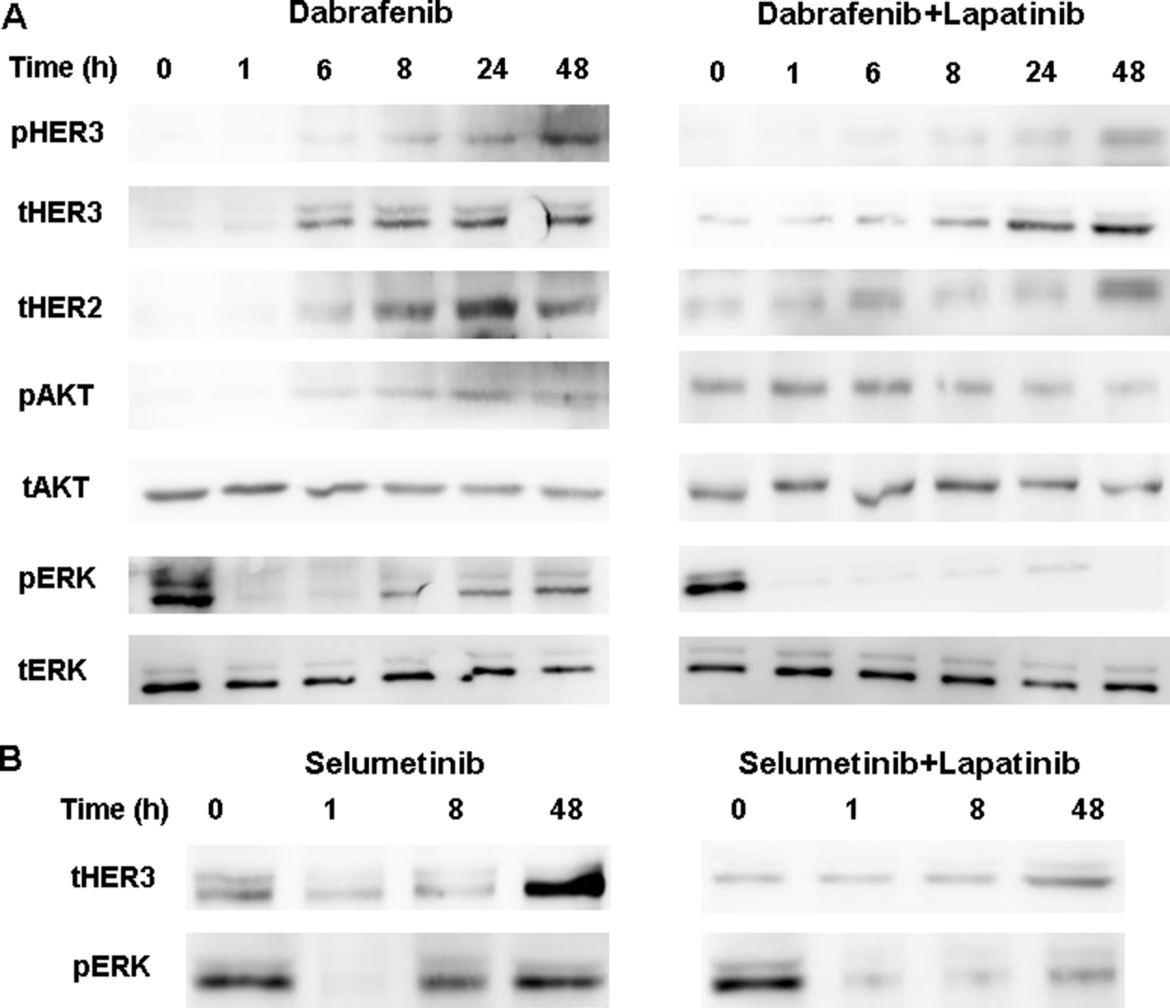
(**A**) Western blot of lysates of BCPAP cells treated with 0.1 μM dabrafenib with or without 1 μM lapatinib for indicated times. Lapatinib blocked the dabrafenib-induced HER3 and AKT phosphorylation and the pERK1/2 rebound. (**B**) BCPAP cells were treated with 2.5 μM selumetib with or without 1 μM lapatinib and collected at 0, 1, 8, and 48 h post-treatment. Lysates were Western blotted for HER3 and ERK.

As shown in Figure 1A, the inhibitory effect of dabrafenib on MAPK signaling pathway in *BRAF*-mutant BCPAP cells was transient, with a MAPK rebound beginning nearly 8h after addition of dabrafenib. The expression of the HER2 and HER3 increased after 6h incubation with dabrafenib, which was accompanied by elevated AKT phosphorylation activity. Lapatinib blocked the superactivated HER2/HER3, AKT and pERK1/2 induced by dabrafenib. In BRAF-mutant K1 cells, HER3 activating and pERK1/2 rebound was also induced with dabrafenib treatment, which was blocked by lapatinib (Supplementary Figure 4).

When the BCPAP cells were incubated with selumetinib, activation of HER and rebound of pERK1/2 was observed and this was prevented via co-incubating with lapatinib (Figure 1B).

### Effect on the expression of iodine- and glucose-handling genes

**Figure 2:**
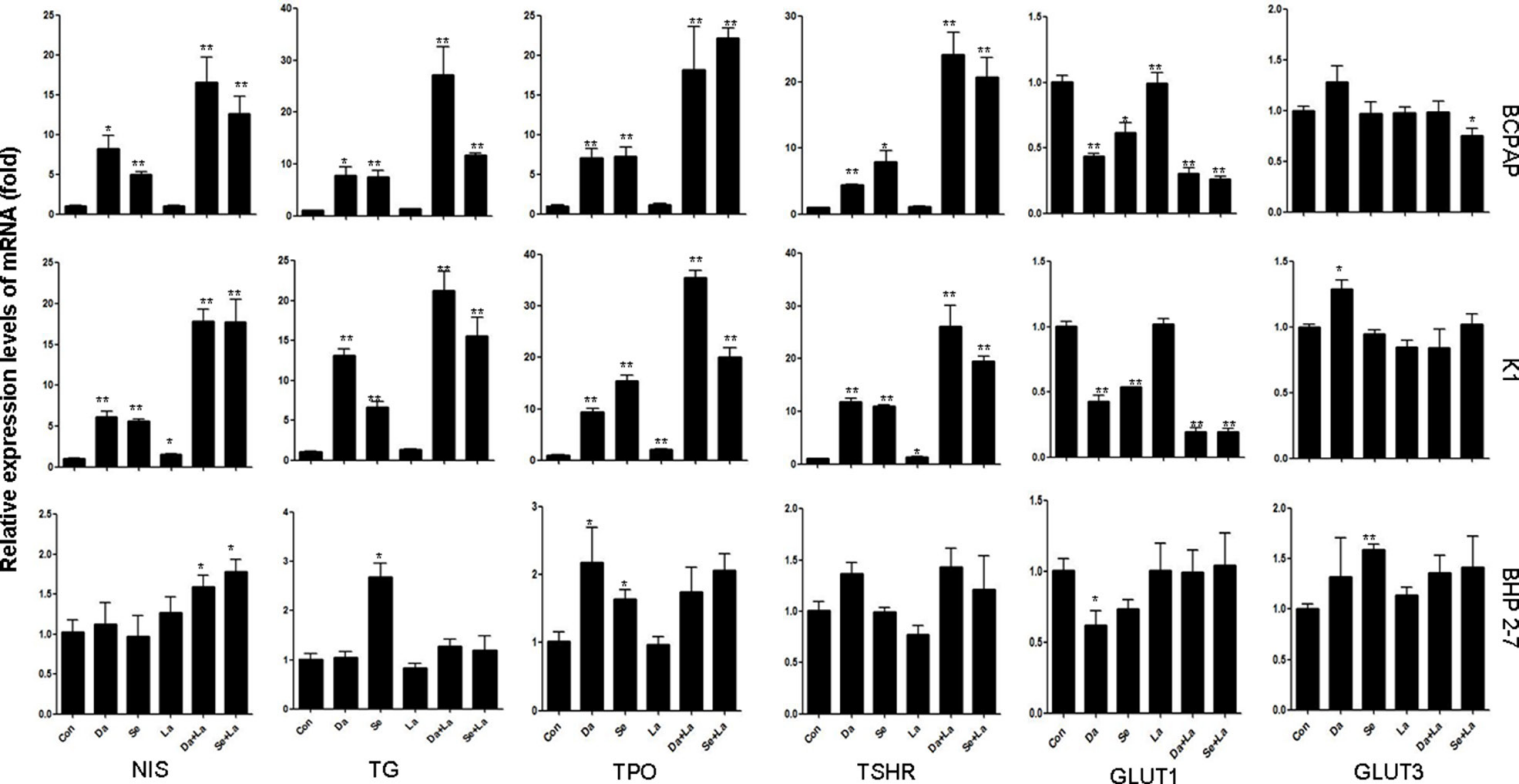
Effects of different treatment on the mRNA levels of sodium iodine symporter (NIS), thyroglobulin (Tg), thyroid peroxidase (TPO), thyroid-stimulating hormone receptor (TSHR); and on glucose transporter isoforms (GLUT1 and GLUT3) genes in BCPAP cells, K1 cells and BHP 2-7 cells. Cells were treated with 0.1 μM dabrafenib/2.5 μM selumetinib and 1 μM lapatinib alone or in combination. Data are presented as means ± SD. *P < 0.05; **P < 0.01 for comparison with control. Con: control (DMSO); Da: dabrafenib; Se: selumetinib; La: lapatinib.

For BCPAP cells and K1 cells, treatment with dabrafenib or selumetinib for 48 hours increased the mRNA levels of iodine-handling genes, including NIS, Tg, TPO, and TSHR (Figure 2). The mRNA levels of GLUT1 reduced, but the mRNA levels of GLUT3 were not significantly changed (Figure 2). In BHP 2-7 cells, this effect was generally modest (Figure 2). Combination of MAPK inhibitor and HER inhibitor lapatinib induced a more robust expression of iodine-handling genes in BCPAP cells and K1 cells while mRNA level of GLUT1 was reduced to a lower extent (Figure 2).

**Figure 3:**
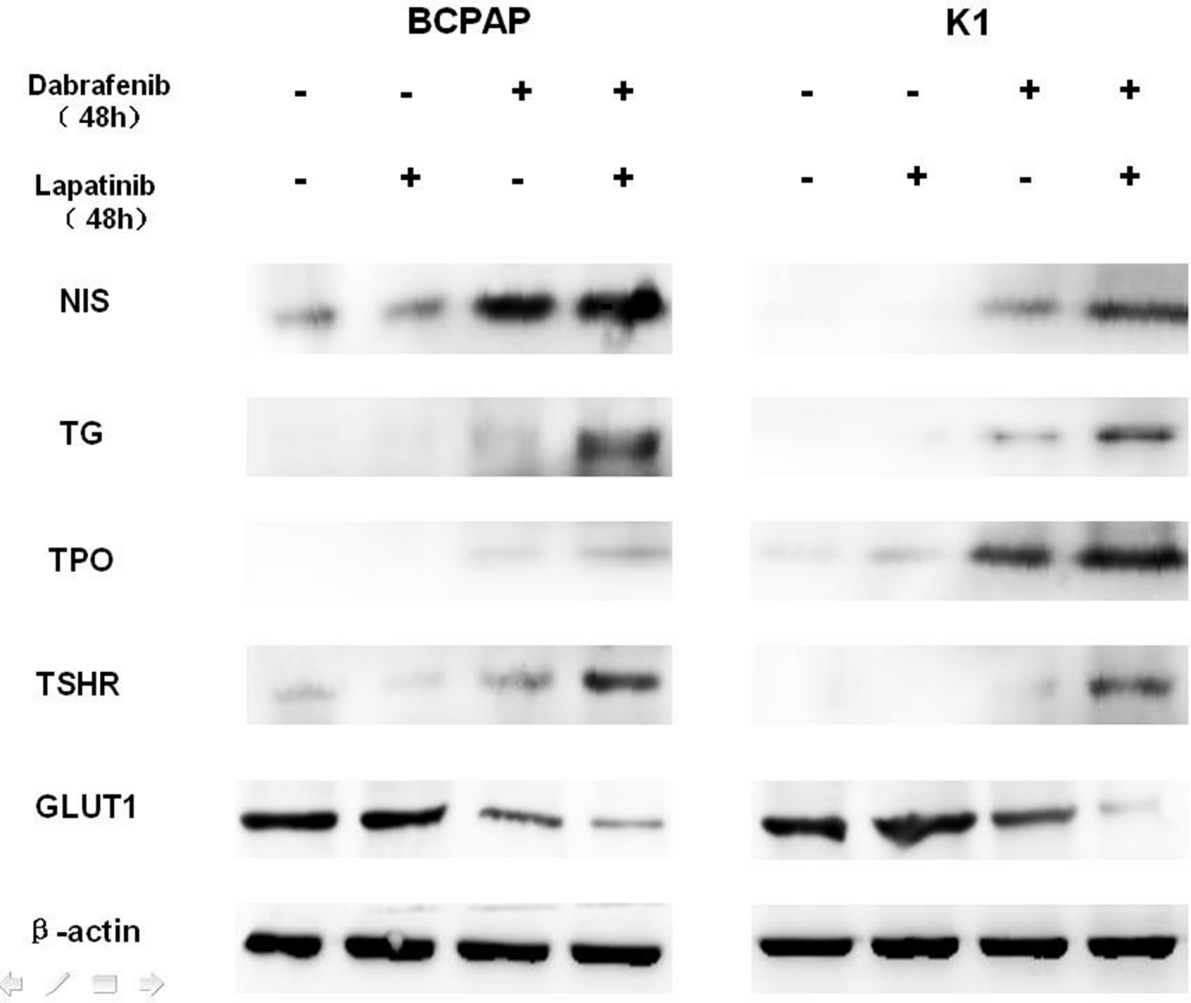
Western blot demonstrating the effects of different treatment on the protein levels of sodium iodine symporter (NIS), thyroglobulin (Tg), thyroid-stimulating hormone receptor (TSHR), thyroid peroxidase (TPO), glucose transporter-1 (GLUT1) in BCPAP (left) and K1 (right) cells. Cells were treated with 0.1 μM dabrafenib alone or in combination with 1 μM lapatinib. β-actin was used as positive control.

Western blot analysis demonstrated that dabrafenib restored the expression of NIS, Tg, TSHR, and TPO, and reduced the expression of GLUT1 (Figure 3) in both BCPAP and K1 cells. More evident effect was observed with dual inhibition of MAPK and HER. For BHP 2-7 cells, however, no significant changes in the expression of glucose and iodine-handling genes were observed (Supplementary Figure 5).

**Figure 4:**
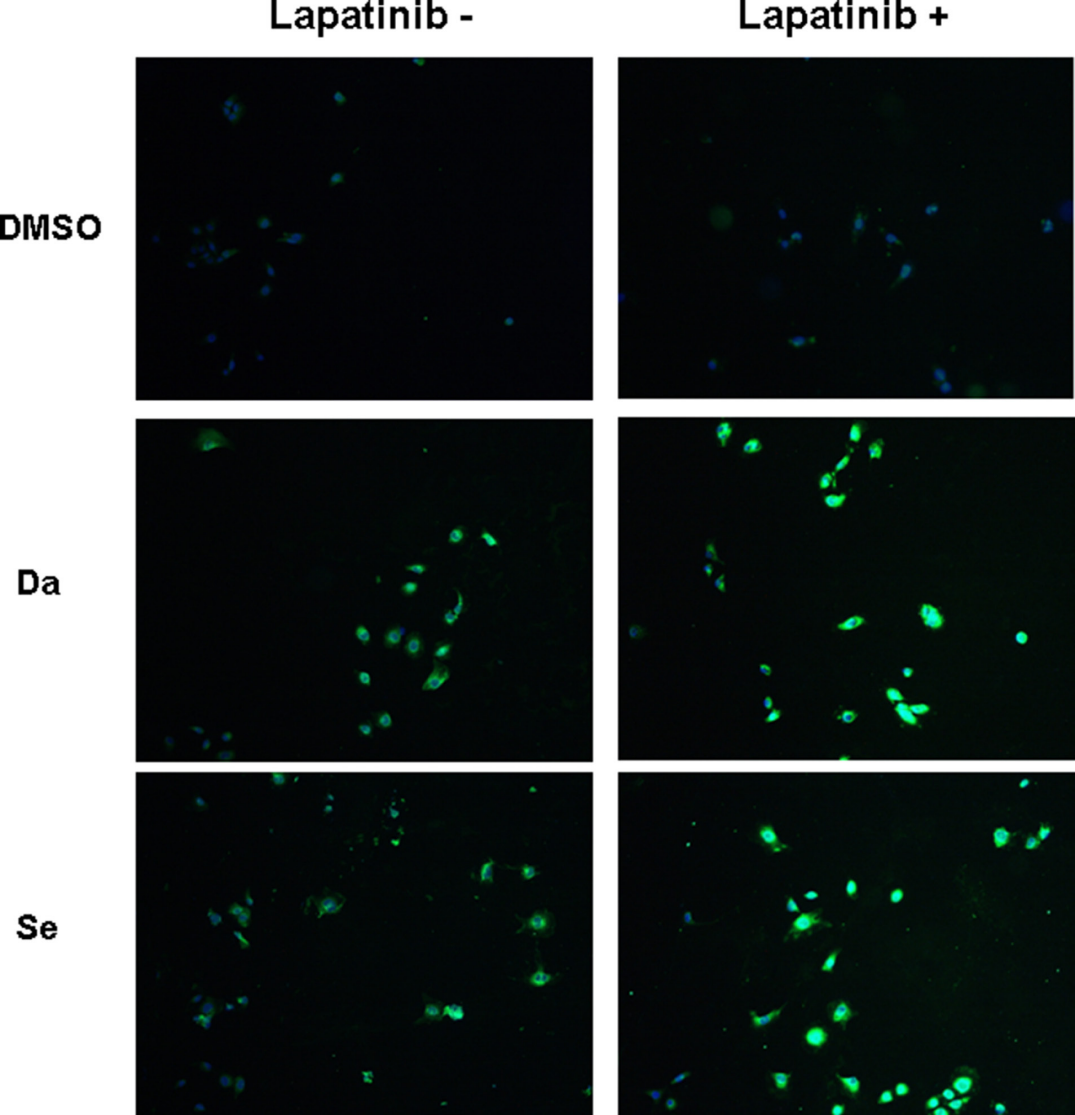
Immunofluorescent microscopic analysis of NIS protein expression in BCPAP cells. Cells were treated with 0.1 μM dabrafenib/2.5 μM selumetinib and 1 μM lapatinib alone or in combination. Double immunofluorescent microscopy was displayed with the blue color representing DAPI nuclear staining and the green color representing NIS staining. NIS staining was negative in the nontreated and lapatinib treated cells. In cells treated with dabrafenib/selumetinib, NIS staining was notable. The most robust expression of NIS was seen in the combined treatment groups. Da: dabrafenib; Se: selumetinib.

NIS protein expression was illustrated by immunofluorescent microscopy. While there was virtually no basal NIS protein expression, NIS staining in the peripheral areas of the cell was notable in BCPAP cells (Figure 4) and K1 cells (Supplementary Figure 6) when treated with dabrafenib or selumetinib, suggesting increased cell membrane localization. NIS was more clearly localized in the peripheral areas of the cell under combined treatment with dabrafenib/selumetinib and lapatinib.

**Figure 5:**
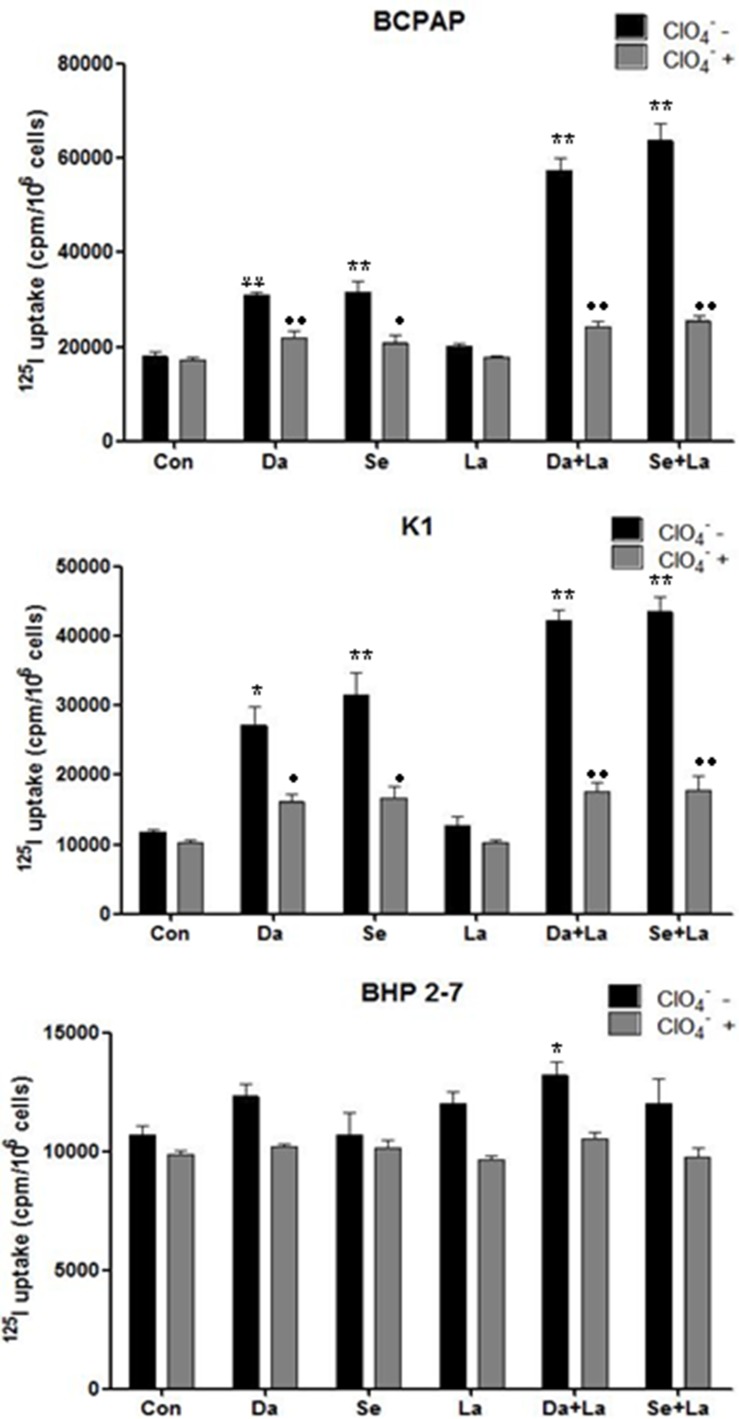
Radioactive iodine uptake in BCPAP cells, K1 cells, and BHP 2-7 cells. Cells were treated with 0.1 μM dabrafenib/ 2.5 μM selumetinib and 1 μM lapatinib alone or in combination. Data are expressed as mean ± SD of values. *P < 0.05, **P < 0.01 compared with untreated cells. ●*P* < 0.05, ● ●*P* < 0.01 compared with NaClO_4_ treated cells. Da: dabrafenib; Se: selumetinib; La: lapatinib.

**Figure 6:**
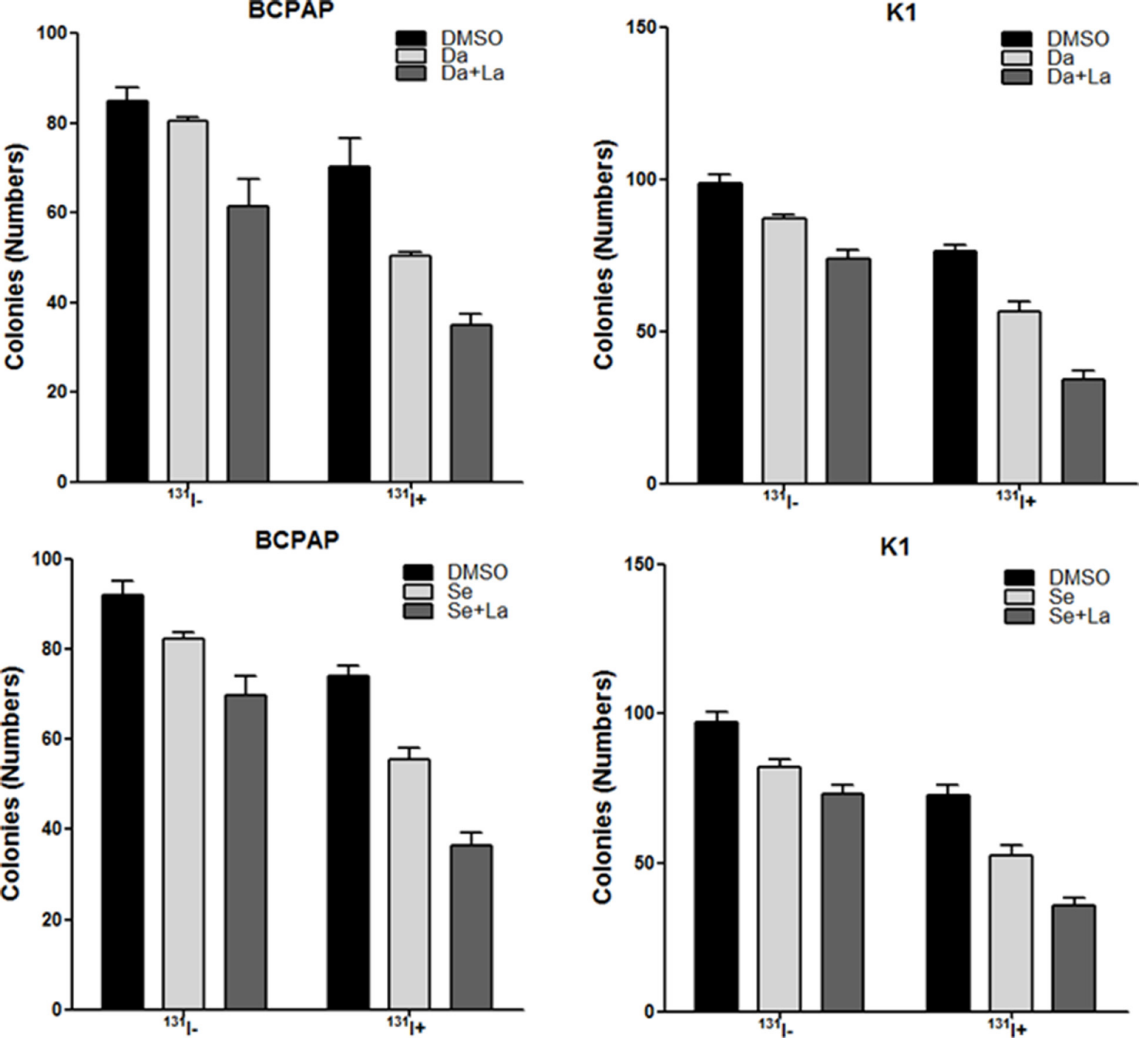
*In vitro* clonogenic assay. Data are represented as number of colonies in BCPAP cells and K1 cells treated with DMSO, 0.1 μM dabrafenib/2.5 μM selumetinib alone or in combination with 1 μM lapatinib with (^131^I+) or without ^131^I (^131^I-). Data are expressed as mean ± SD. Da: dabrafenib; Se: selumetinib; La: lapatinib.

### Radioiodine uptake and efflux assay

As shown in Figure 5, radioiodine uptake was 1.72-fold higher (*P* < 0.01) in BCPAP cells and 1.64-fold higher (*P* < 0.05) in K1 cells when incubated with dabrafenib compared with nontreated cells. Combined treatment with dabrafenib and lapatinib induced 3.20-fold higher (*P* < 0.01) and 3.73-fold higher (*P* < 0.01) radioiodine uptake in BCPAP cells and K1 cells respectively. When treated with selumetinib, radioiodine uptake was 1.72-fold higher (*P* < 0.01) in BCPAP cells and 2.69-fold higher (*P* < 0.01) in K1 cells compared with nontreated cells. Combination of selumetinib and lapatinib induced 3.55-fold higher (*P* < 0.01) and 4.04-fold higher (*P* < 0.01) radioiodine uptake in the two cell lines respectively. Iodine uptake was specifically dependent on NIS because it was blocked by NaClO_4_. Radioiodine uptake of BHP 2-7 cells increased when using combined treatment with dabrafenib and lapatinib (*P* = 0.022), while did not increased with other treatment.

We also evaluate the ability of BCPAP cells and K1 cells to retain iodine under single-drug or combined treatment (Supplementary Figure 7). There was a rapid efflux of radioactivity from the non-treated BCPAP and K1 cells (t_1/2_ = 6.74 min and t_1/2_ = 6.22 min respectively), iodine retention was not significantly prolonged in the dabrafenib treated group (t_1/2_ = 8.53 min in BCPAP cells and t_1/2_ = 7.09 min in K1 cells, *P* = 0.194 and *P* = 0.118, respectively) or in selumetinib treated group (t_1/2_ = 8.20 min in BCPAP cells and t_1/2_ = 7.66 min in K1 cells, *P* = 0.108 and *P* = 0.149, respectively). Nor was retention time prolonged in combined treatment groups (t_1/2_ = 9.00 min in BCPAP cells and t_1/2_ = 8.57min in K1 cells, *P* = 0.083 and *P* = 0.057, respectively in dabrafenib & lapatinib group and t_1/2_ = 9.09min in BCPAP cells and t_1/2_ = 7.75 min in K1 cells, *P* = 0.065 and *P* = 0.332, respectively in selumetinib & lapatinib group).

### *In vitro* clonogenic assay

In ^131^I treated BCPAP cells and K1 cells, compared to DMSO treated controls, the numbers of colonies were significantly smaller in dabrafenib treated groups (*P* = 0.034 for BCPAP cells and *P* = 0.009 for K1 cells) and dabrafenib & lapatinib treated groups (*P* = 0.006 for BCPAP cells and *P* = 0.000 for K1 cells). Further, the numbers of colonies were significantly smaller in dabrafenib & lapatinib treated groups compared to that in dabrafenib treated groups (*P* = 0.005 for BCPAP cells and *P* = 0.006 for K1 cells) (Figure 6).

In ^131^I treated BCPAP cells and K1 cells, compared to DMSO treated controls, the numbers of colonies decreased significantly in dabrafenib treated groups (*P* = 0.005 for BCPAP cells and *P* = 0.013 for K1 cells) and dabrafenib & lapatinib treated groups (*P* = 0.001 for BCPAP cells and *P* = 0.001 for K1 cells). Further, dabrafenib combined with lapatinib induced a significant reduction in the numbers of colonies compared to that in dabrafenib treated groups (*P* = 0.007 for BCPAP cells and *P* = 0.017 for K1 cells) (Figure 6).

## DISCUSSION

Silencing of NIS, along with other iodine-handling genes results in resistance to radioiodine treatment, which is associated with activation of MAPK pathway [4]. Recent progresses in both preclinical and clinical study have provided promises for blockading the MAPK pathway to restore radioiodine avidity [6–10]. In our previous study, sorafenib and carbozatinib have been demonstrated to blockade the MAPK pathway and increase radioiodine uptake in PTC cells [6]. However, multikinase inhibitors have worse patient tolerability than BRAF/MEK inhibitors [16–18], so redifferentiation therapy with these BRAF/MEK inhibitors to facilitate treatment with radioiodine represents a better choice for patients with RR-DTC. Unfortunately, in clinical trials with small sample size, rediffereniation therapy with MEK or BRAF inhibitors failed to meet high expectation [9, 10]. Novel strategies to augment the effect of BRAF/MEK inhibitor-induced redifferentiation are urgently needed.

Recently, much work has been done on exploring mechanisms of resistance to BRAF inhibitors in various cancers including thyroid cancer [12–14, 16]. Fagin and colleagues reported that BRAF/MEK inhibitor treated thyroid cells showed HER activating, thereby increasing the activation of both the MAPK and PI3K pathways. It was further demonstrated that combination with HER inhibitor lapatinib prevented MAPK rebound and sensitized *BRAF*-mutant thyroid cancer cells to BRAF or MEK inhibitors [15]. This study provides a rationale for combining ERK pathway antagonists with inhibitors of feedback reactivated HER signaling in thyroid cancer, yielding a sustained and enhanced redifferentiation effect.

In the present study, we utilized *in vitro* model to evaluate the efficacy of dabrafenib /selumetinib in combination with lapatinib, a drug with acceptable adverse effect profiles in patients with breast and head/neck cancer [19], as a potential combined redifferentiation therapeutic strategy. Using such combination strategy in *BRAF*^V600E^-positive PTC cells, the expression of iodine-handling genes in thyroid cancer cells increased to a greater extent, localization of NIS to the cell membrane was greater promoted, and radioiodine uptake and toxicity were augmented to a higher level. In line with prior study using vemurafenib and lapatinib [15], MAPK rebound and PI3K pathway activation after dabrafenib/selumetinib treatment was detected in a time dependent manner, and addition of lapatinib to the BRAF/MEK inhibitor prevented activation of MAPK and PI3K pathways, which may be the mechanism underlying this combined redifferentiation effect. Our data suggest that combined therapy using HER inhibitor and BRAF/MEK inhibitor can produce more significant redifferentiating effect on thyroid cancer cells than single drug treatment with BRAF/MEK inhibitor alone, since MAPK rebound during BRAF/MEK inhibitor treatment can be effectively prevented. Our study provides basis for *in vivo* and clinical studies using combination of dabrafenib/selumetinib and lapatinib in conjunction with ^131^I therapy, which may become a promising therapeutic regimen for RR-DTC, particularly in *BRAF* ^V600E^-positive cases.

Iodine accumulation in thyroid follicle cells involves two steps of transport, basolateral uptake and apical efflux, which are both important factors for cytotoxic effect of ^131^I [20]. The efflux of iodine across the apical membrane is mediated by pendrin, SLC5A8, ClCn5 and Anoctamin 1, etc. Iodine efflux or retention represents a team effort by various apical membrane proteins [21–25]. Previous studies have revealed regulation mechanisms of several apical membrane iodine transporters: pendrin membrane abundance is regulated by TSH [21, 26]; silencing of SLC5A8 is secondary to the constitutive activation of the MAPK pathway [26, 27]. In our study, however, neither dabrafenib/selumetinib treatment nor combined treatment significantly changed radioiodine retention time in thyroid cancer cells, although MAPK pathway was blocked by these treatments. It maybe due to that MAPK inhibitor alone is not sufficient to alter the activities of all the apical membrane proteins.

An inverse relationship between ^131^I and ^18^F-FDG uptake (“flip-flop phenomenon”) was described for thyroid cancers during dedifferentiation [5]. A molecular mechanism underlying ^18^F-FDG uptake in poorly differentiated thyroid cancers is that dedifferentiation in thyroid cancer is accompanied by GLUT1 upregulation [3, 5]. In our previous study, the expression of GLUT1 and GLUT3 and the ^18^F-FDG uptake were significantly decreased in BHP 2-7 cells harboring RET/PTC rearrangement after redifferentiation therapy with either sorafenib or carbozatinib, indicating that monitoring efficacy of redifferentiation therapy using ^18^F-FDG PET is possible [6]. The present study demonstrated that the expression levels of GLUT1 in DTC cells decreased significantly after BRAF/MEK inhibition and was even lower after dual inhibition of BRAF/MEK and HER, once again providing rationale for monitoring efficacy of redifferentiation therapy using ^18^F-FDG PET. However, GLUT3 expression level was not significantly changed in any of the treated groups, which is different from the trend in our previous study, while this was consistent with Perrier’s findings that GLUT1 is upregulated during thyroid carcinogenesis and other GLUTs were statistically unchanged [28].

Previous studies demonstrated *BRAF*^V600E^-dependent inhibition in thyroid cancer cells by BRAF inhibitors [29, 30]. Compared to *BRAF* wild-type BHP 2-7 cells, BRAF inhibitor dabrafenib preferentially inhibited ERK signaling and thyroid cancer cell proliferation, arrested cell circle, and regulated iodine and glucose-handling gene expression in *BRAF*^V600E^-positive PTC cells. A possible explanation of this phenomenon is that BRAF inhibitor binding activates wild-type RAF isoforms by inducing dimerization, membrane localization and interaction with RAS-GTP, which ultimately activates the MAPK pathway [31]. It is noteworthy that although MEK inhibitor also exhibited preferentially inhibition of *BRAF*^V6^00E-positive PTC cells owing to its target just downstream of BRAF, selumetinib lacked efficacy on *BRAF* wild-type BHP 2-7 cells for the regulation of cell proliferation, apoptosis, cell cycle and metabolism, which is probably due to persistent activating of PI3K/AKT pathway, negating its inhibitory effect on ERK signaling [32, 33].

It must be pointed out that although dabrafenib, selumetinib and lapatinib have acceptable adverse effects. In clinical use, patients receiving the combination therapy may require more dose modifications than did those receiving monotherapy. Interrupting treatment at the first sign of severe side effect is critical. To our comfort, however, previous study comparing combined BRAF and MEK inhibition to BRAF inhibition showed no significant between-group difference in the frequency of adverse events, including grade 3 and 4 toxic effects [34].

In summary, our *in vitro* study suggests that BRAF/MEK inhibitor-induced redifferentiation effect in PTC cells harboring *BRAF*^V600E^ can be promoted by HER inhibitor via effectively preventing MAPK rebound. It provides basis for *in vivo* and clinical studies using combination of dabrafenib/selumetinib and lapatinib in conjunction with ^131^I therapy, which may become a potential therapeutic regimen for RR-DTC, particularly in *BRAF*^V600E^-positive patients.

## MATERIALS AND METHODS

### Cell culture and agents

The K1 cell line was obtained from health protection agency culture collection, BCPAP cell line was purchased from Chinese Academy of Science. BHP 2-7 cell line was kindly provided by Prof. Jerome M. Hershman. All cell lines were maintained at 37°C and 5% CO_2_ in humidified atmosphere and grown in RPMI 1640 growth media supplemented with 10% fetal bovine serum (GIBCO). Under these culture conditions, cells were treated with dabrafenib, selumetinib and lapatinib (MCE) individually or in combination for indicated times. Dimetylsulfoxide (DMSO) was used in parallel as vehicle control.

### Oncogene analysis and cell proliferation assay

Total RNAs were prepared from cells cultured for oncogene analysis using RNeasy kit (Qiagen). Primer sequences are displayed in Supplementary Table 1. The Cell Counting Kit-8 (DOJINDO) was used to detect cell proliferation after cells were cultured with increasing concentrations of dabrafenib, selumetinib and lapatinib alone or in combination for 24 h, 48 h, and 72 h. IC_50_ was calculated using Prism 5.0 (GraphPad Software).

### Cell cycle analysis

Cells (3.0×10^5^) were grown in 25 cm^2^ flasks overnight and incubated with dabrafenib at 0.1 μM, selumetinib at 2.5 μM and lapatinib at 1 μM individually or in combination or DMSO for 24 h, and subsequently used for cell cycle analysis. The samples were analyzed by flow cytometry (Becton Dickinson, USA).

### RNA extraction and real-time quantitative RT-PCR analysis

Cells (3.0×10^5^) were seeded in 25 cm^2^ flasks and then incubated with dabrafenib at 0.1 μM, selumetinib at 2.5 μM and lapatinib at 1 μM individually or in combination or DMSO for 48 h. Total RNA was isolated from cells using RNeasy kit (Qiagen), Total RNA (1 μg) was converted to cDNA on an iCycler Thermal Cycler (Bio-Rad Laboratories) using QuantiTect Reverse Transcription Kit (Qiagen). Realtime quantitative RT-PCR analysis was performed on an ABI Prism 7900HT Sequence Detector (Applied Biosystems) using SYBR Green MasterMix(Qiagen). 18S was run in parallel to standardize the input cDNA. Primer design was performed using Primer Express 2 [6].

### Western blotting assay

Cells were lysed in RIPA buffer. Equal amounts of total protein were resolved by SDS-PAGE, transferred to PVDF membranes (Millipore), and immunoblotted with the indicated primary antibodies. Membranes were hybridized with the following primary antibodies: p-Erk1/2, Erk1/2, p-HER3/ErbB3, HER3/ErbB3, HER2/ErbB2, p-AKT, AKT (Cell Signaling Technology), NIS, Tg, TPO, TSHR, GLUT1, and GLUT3 (Protein tech). Membranes were then hybridized with species-specific HRP-conjugated antibodies. Bands were visualized with Potent ECL kit (Beyotime).

### Immunofluorescent localization of NIS

Cells (2.0×10^4^) were seeded in 6-well chamber slides. After 72 h of incubation with specific inhibitors, cells were fixed in paraformaldehyde. Cells were then incubated in succession with rabbit anti-NIS (1:100; Protein tech), Alexa Fluor 488-conjugated Goat anti-rabbit IgG secondary antibody (1:100, Thermo) and DAPI. Fluorescent microscopic examination was conducted to monitor NIS expression (Nikon Corporation, Tokyo, Japan).

### Iodine uptake and efflux assay

Cells (1.5 × 10^5^) were seeded in 6-well plates and then incubated with dabrafenib at 0.1 μM, selumetinib at 2.5 μM and lapatinib at 1 μM individually or in combination or DMSO for 48 h. For iodine uptake assay, one well was counted for cell number for each group, and the remaining wells were incubated in 1mL serum-free RPMI 1640 containing 2 μCi Na^125^I. Following incubation at 37°C for 1 h, the medium containing Na^125^I was removed and the cells were washed twice with PBS. Then the cells were lysed with 0.3 mol/L sodium hydroxide on ice. Cell-associated radioiodine was measured with a gamma counter. To avoid any unspecific iodine uptake, control cells were pre-incubated with 300 μM NaClO_4_ for 30 min and then treated with Na^125^I as described above.

For iodine efflux assay, cells (4 × 10^4^) were seeded in 12-well plates and prepared as those in iodine uptake assay. Cells were incubated in 500 μL serum-free RPMI 1640 containing 1 μCi of Na^125^I at 37°C for 1 h. Cells were washed twice with PBS, and then 500 μL serum-free RPMI 1640 was added per well. The serum-free RPMI 1640 was replaced every 5 min for 30 min and the radioactivity of ^125^I in the collected medium was measured with a gamma counter. After the last time point, trapped ^125^I was measured. Total radioactivity at the beginning of the efflux study (100%) was calculated by summing radioactivity of collected medium at different time point and final radioactivity of cells.

### *In vitro* clonogenic assay

Cells (4 × 10^2^) were seeded into 6-well plates and left 48 h to attach. 48 h after treatment with targeted drugs, a clonogenic assay was performed. Briefly, drug-containing medium was discarded and cells were washed twice in PBS; the medium was then replaced with 1 mL of regular culture medium in the presence or absence of 20 μCi Na^131^I for 6 h. At the end of the treatment the radioactive medium was discarded, and cells were incubated in regular culture medium for 7 days. Finally, cells were fixed in methanol and stained with crystal violet and the number of macroscopic colonies was counted [35].

### Statistical analysis

All the experiments were similarly done at least three times. The cell cycle assay data were compared using the chi square test. The data from the RT-PCR assay, radioiodine uptake and efflux assay were compared using the independent-samples t test. All statistical analyses were performed using a statistical software program (SPSS, version 17.0; SPSS, Inc. Chicago, IL, USA). Significance was defined as *P* < 0.05.

## SUPPLEMENTARY MATERIALS FIGURES


