# Supplementary material for: HER inhibitor promotes BRAF/MEK inhibitor-induced redifferentiation in papillary thyroid cancer harboring BRAFV600E
Source: Oncotarget. 2017 Feb 28;8(12):19843–54. doi: 10.18632/oncotarget.15773 (PMC5386727; doi:10.18632/oncotarget.15773)
Supplement: Supplementary file 1 [file oncotarget-08-19843-s001.pdf]

HER inhibitor promotes BRAF/MEK inhibitor-induced redifferentiation in papillary thyroid cancer harboring *BRAF*^{V600E}

Supplementary Materials

Oncogene analysis

Total RNAs were prepared from cells cultured using RNeasy kit (Qiagen). RNA (2 μg) was reverse-transcribed into 40 μL cDNA using QuantiTect Reverse Transcription Kit (Qiagen). Primer design was performed using Primer Express 2, and the sequences are presented in supplemental table 1. Reactions were performed in 50 μL of a PCR solution containing 4 μL cDNA, 1 μM primer with buffers from Hotstar Taq^{TM} plus DNA polymerase kit (Qiagen). PCR products were visualized by electrophoresis in 2% agarose gel. Products were submitted to direct sequencing. Mutations of *NRAS*, *BRAF*, *PIK3CA* and *RET/PTC1* (primer sequence was seen in supplemental table 1) were confirmed by sequencing of antisense strands. The passage of the cells at which these evaluations had been performed is 15-20. Three replicate experiments were performed.

Cell cycle analysis

Cells (3.0×10^5) were grown in 25 cm^2 flasks overnight in serum free RPMI 1640 and incubated with dabrafenib at 0.1 μM, selumetinib at 2.5 μM and lapatinib at 1 μM individually or in combination or DMSO for 24 h, then washed twice with cold phosphate-buffered saline (PBS), and transferred by trypsinization to a 15 mL tube containing 300 μL cold PBS and 700 μL cold dehydrated alcohol. After cells were washed, DNA was stained with 50 mg/mL propidium iodide (PI) solution (0.02 mg/mL RNase A, 1.0 mg/ml sodium citrate dihydrate, 0.1 mg/mL PI, and 0.3% Triton X-100) at room temperature for 30 min protected from light. Fluorescence-activated cell sorting (FACS) was performed using flow cytometry (Becton Dickinson, USA). Cell cycle analysis was performed by ModFit LT software (Verity Software House, Inc.).

Western blotting assay

Cells were lysed in RIPA buffer with Protease Inhibitor Cocktail (SIGMA). Cellular proteins were collected by centrifugation of lysates at 4°C for 20 minutes. Equal amounts of total protein (40ug) were resolved by 6%-12% SDS-PAGE, transferred to PVDF membranes (Millipore), and immunoblotted with the indicated primary antibodies. Membranes were hybridized with the following primary antibodies: p-Erk1/2, 1:2000,; Erk1/2, 1:2000,; p-HER3/ErbB3, 1:1000,; HER3/ErbB3, 1:2000; p-AKT, 1:2000; AKT, 1:2000 (Cell Signaling Technology). NIS, Tg, TPO, TSHR, GLUT1, and GLUT3 were purchased from(Protein tech). Membranes were hybridized with species-specific HRP-conjugated antibodies, (goat anti-mouse IgG-HRP, 1:2000, and or goat anti-rabbit IgG-HRP, 1:2000 (, Santa Cruz Biotechnology Inc.). Bands were visualized with Potent ECL kit (Beyotime).

Supplementary Table 1: Primer sequences of *NRAS, BRAF, PIK3CA,* and *RET/PET1*

Primer	Sequence
NRAS	forward 5′-agc ttg agg ttc ttg ctg g-3′
	reverse 5′-atc cgc aaa tga ctt gct att a-3′
BRAF	forward 5′-atc cac aga gac ctc aag agt aat a-3′
	reverse 5′-tcc tcg tcc cac cat aaa a-3′
PIK3CA	forward 5′-gga aaa ata tga caa aga aag cta-3′
	reverse 5′-gta tgg taa aaa cat gct gag atc-3′
*RET/PET*1	forward 5′-aga tag agc tgg aga cct aca aac-3′
	reverse 5′-ggt ggt tga cct gct tca g-3′

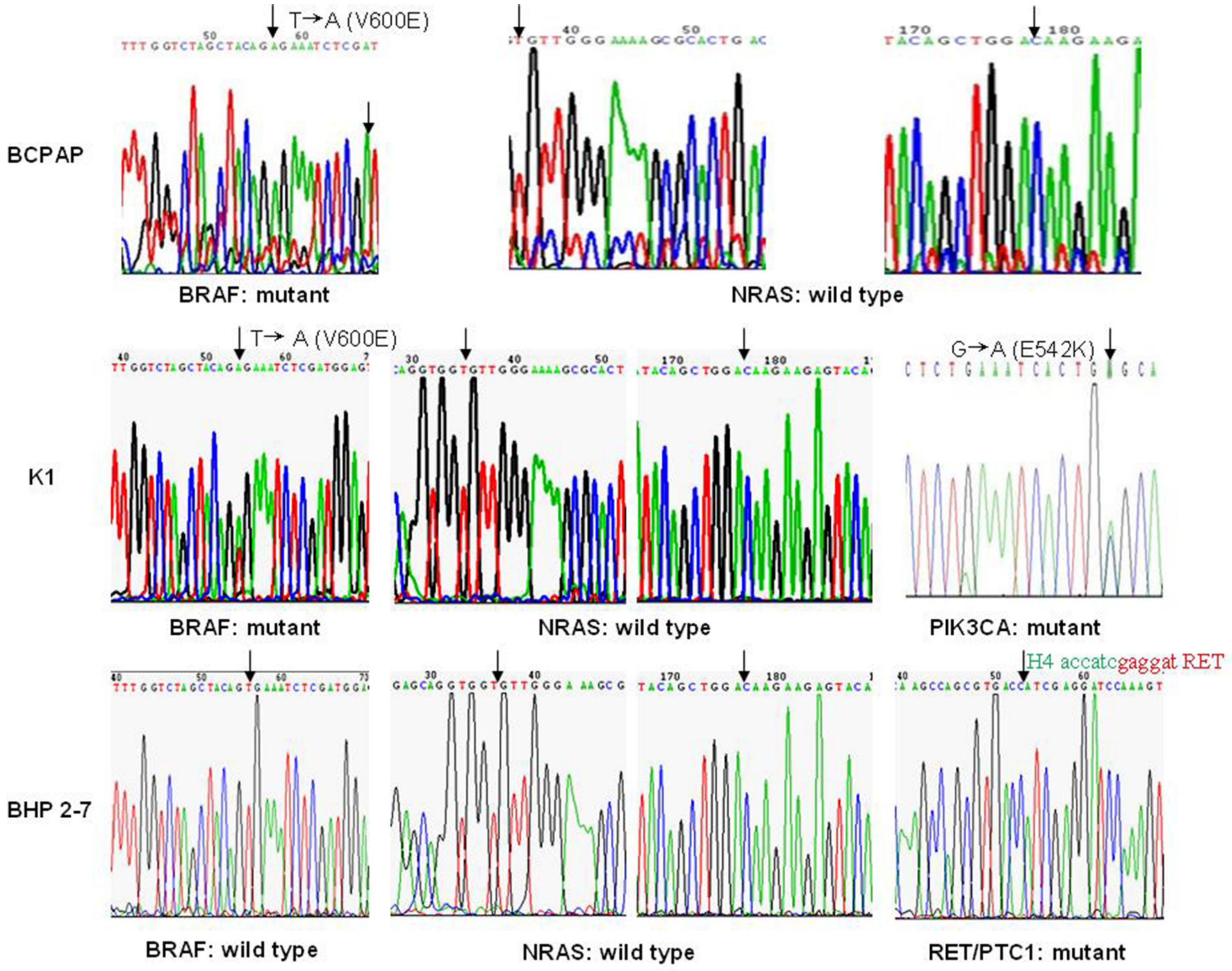

Supplementary Figure 1: Sequence chromatogram of PCR product from BCPAP, K1 and BHP 2-7 cells. BCPAP cells displaying *BRAF*^{V600E} mutant banding pattern a with wild type gene of *NRAS*. K1 cells displaying *BRAF*^{V600E} mutant banding pattern and *PIK3CA* mutant banding pattern with wild type gene of *NRAS*. BHP 2-7 cells displaying *RET/PTC1* rearrangement banding pattern with wild type gene of *BRAF* and *NRAS*.

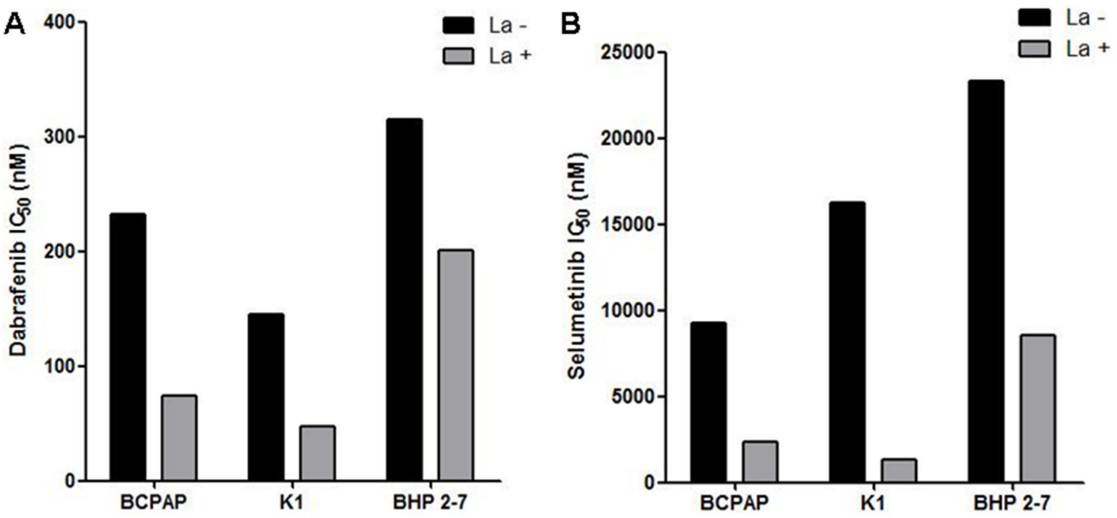

Supplementary Figure 2: (**A**) bars represent IC_{50} values for dabrafenib (with or without 1μM lapatinib co-incubation) in BCPAP cells, K1 cells and BHP 2-7 cells. (**B**) bars represent IC_{50} values for selumetinib (with or without 1μM lapatinib co-incubation) in BCPAP cells, K1 cells and BHP 2-7 cells. La: lapatinib.

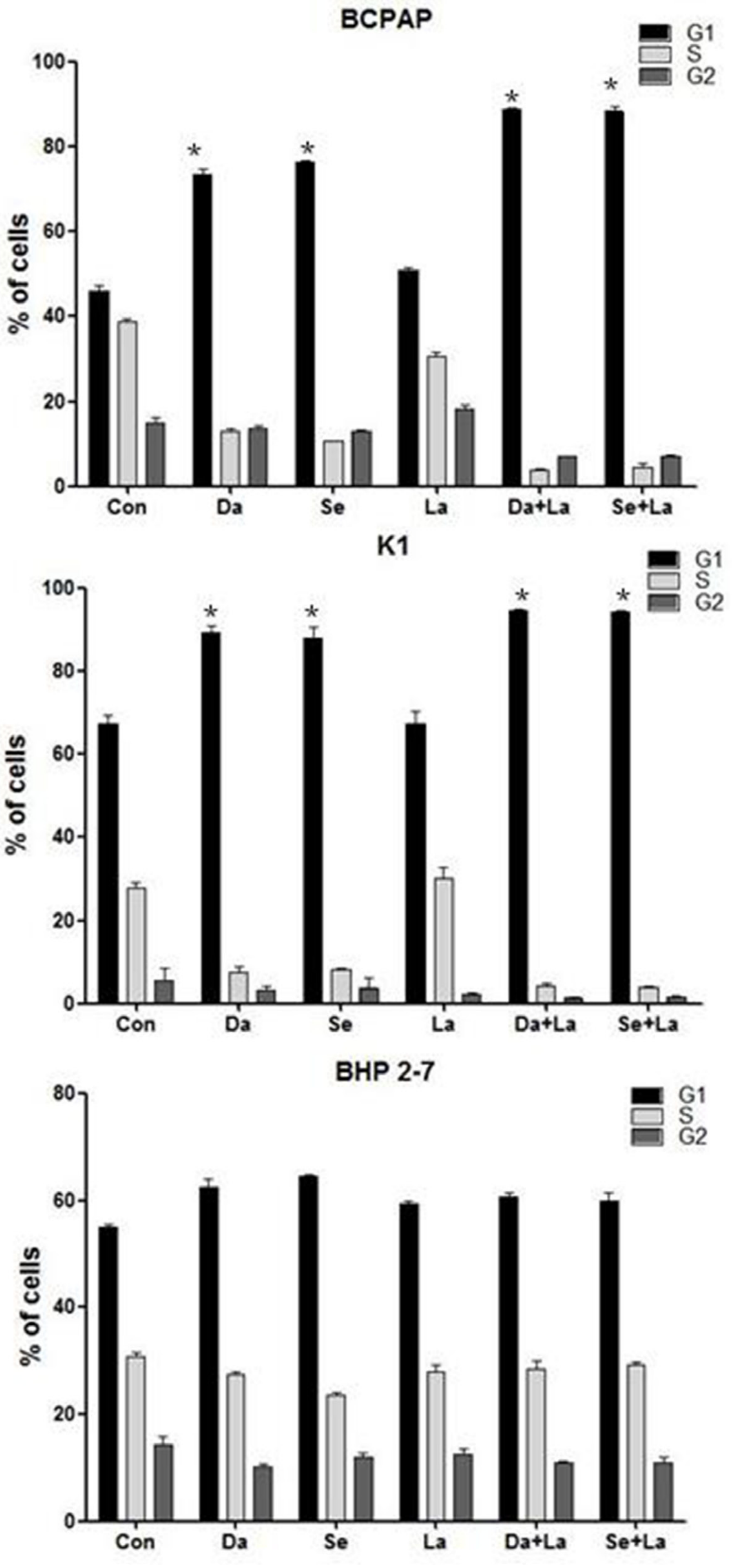

Supplementary Figure 3: Cell cycle analysis of BCPAP cells, K1 cells and BHP 2-7 cells under different treatments. BCPAP cells (**A**), K1 cells (**B**) and BHP 2-7 cells (**C**) were treated with 0.1 μM dabrafenib/2.5 μM selumetinib and 1 μM lapatinib alone or in combination. Cells treated with dimethylsulfoxide (DMSO) were used as the control. After flow cytometry, the percentage of cells in the G1, G2, and S phases were shown. Da: dabrafenib; Se: selumetinib; La: lapatinib.

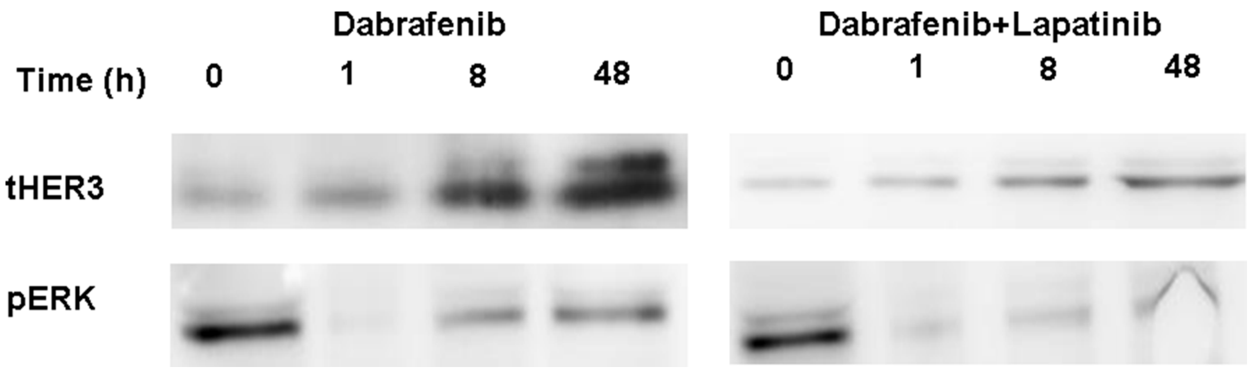

Supplementary Figure 4: Western blot of HER3 and ERK in K1 cells. Cells were treated with 0.1 μM dabrafenib with or without 1 μM lapatinib and collected at 0, 1, 8, and 48 h post-treatment.

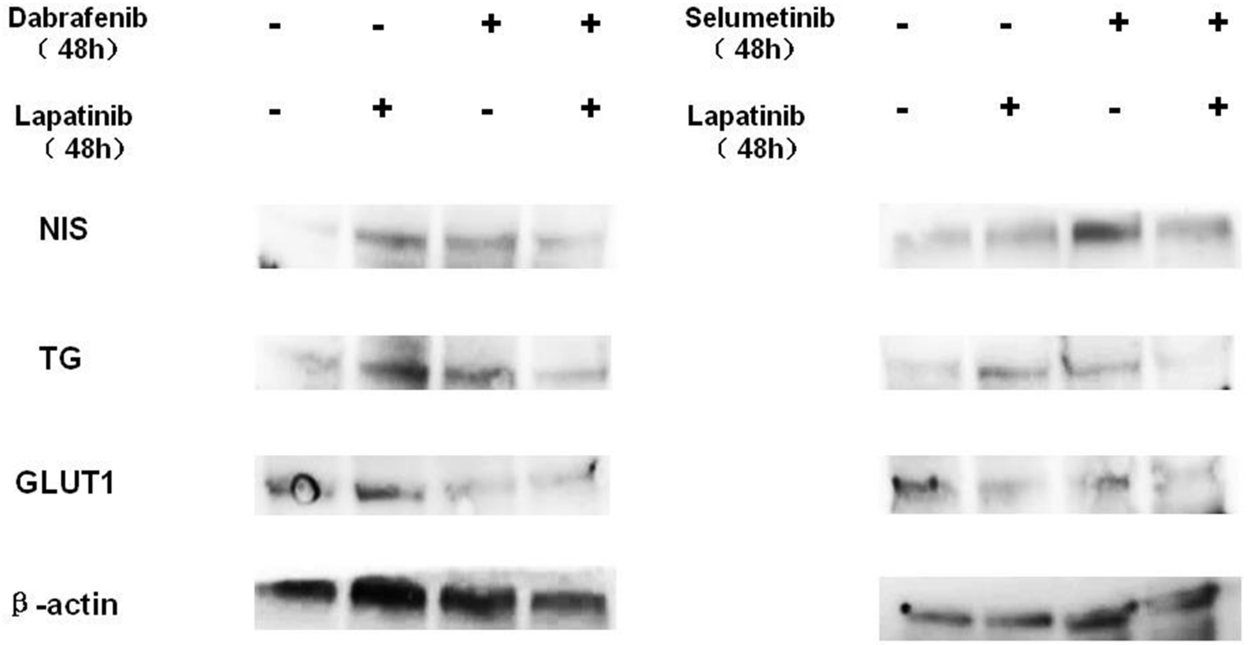

Supplementary Figure 5: Western blot demonstrating the effects of different treatment on the protein levels of sodium iodine symporter (NIS), thyroglobulin (Tg), glucose transporter-1 (GLUT1) in BHP 2-7 cells. Cells were treated with 0.1 μM dabrafenib/ 2.5 μM selumetinib alone or in combination with 1 μM lapatinib. β-actin was used as positive control.

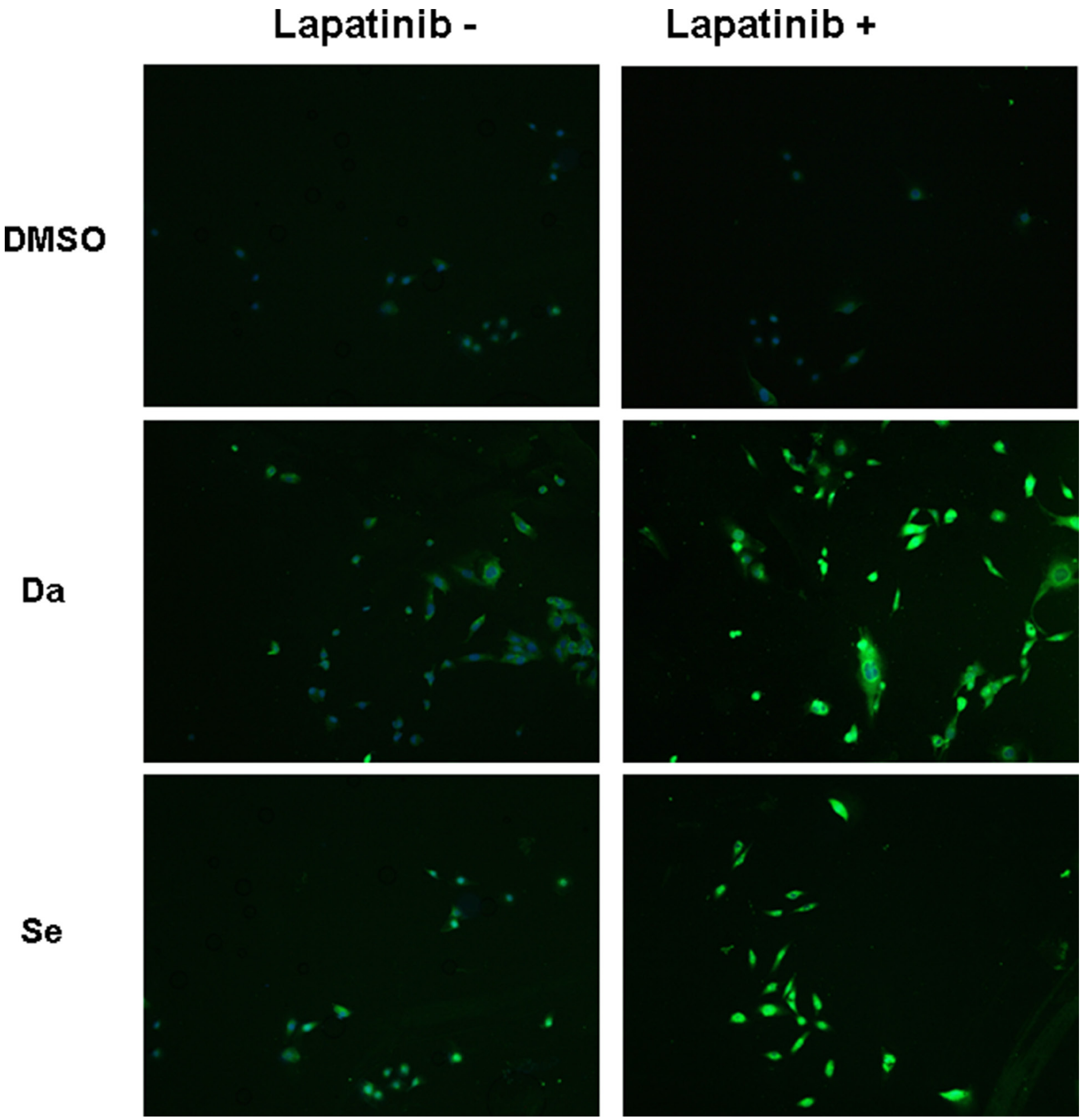

Supplementary Figure 6: Immunofluorescent microscopic analysis of NIS protein expression in K1 cells. Cells were were treated with 0.1 µM dabrafenib/2.5 µM selumetinib and 1 µM lapatinib alone or in combination. Double immunofluorescent microscopy was displayed with the blue color representing DAPI nuclear staining and the green color representing NIS staining. NIS staining was negative in the nontreated and lapatinib treated cells. In cells treated with dabrafenib/selumetinib, NIS staining was notable. The most robust expression of NIS was seen in the combined treatment groups. Da: dabrafenib; Se: selumetinib.

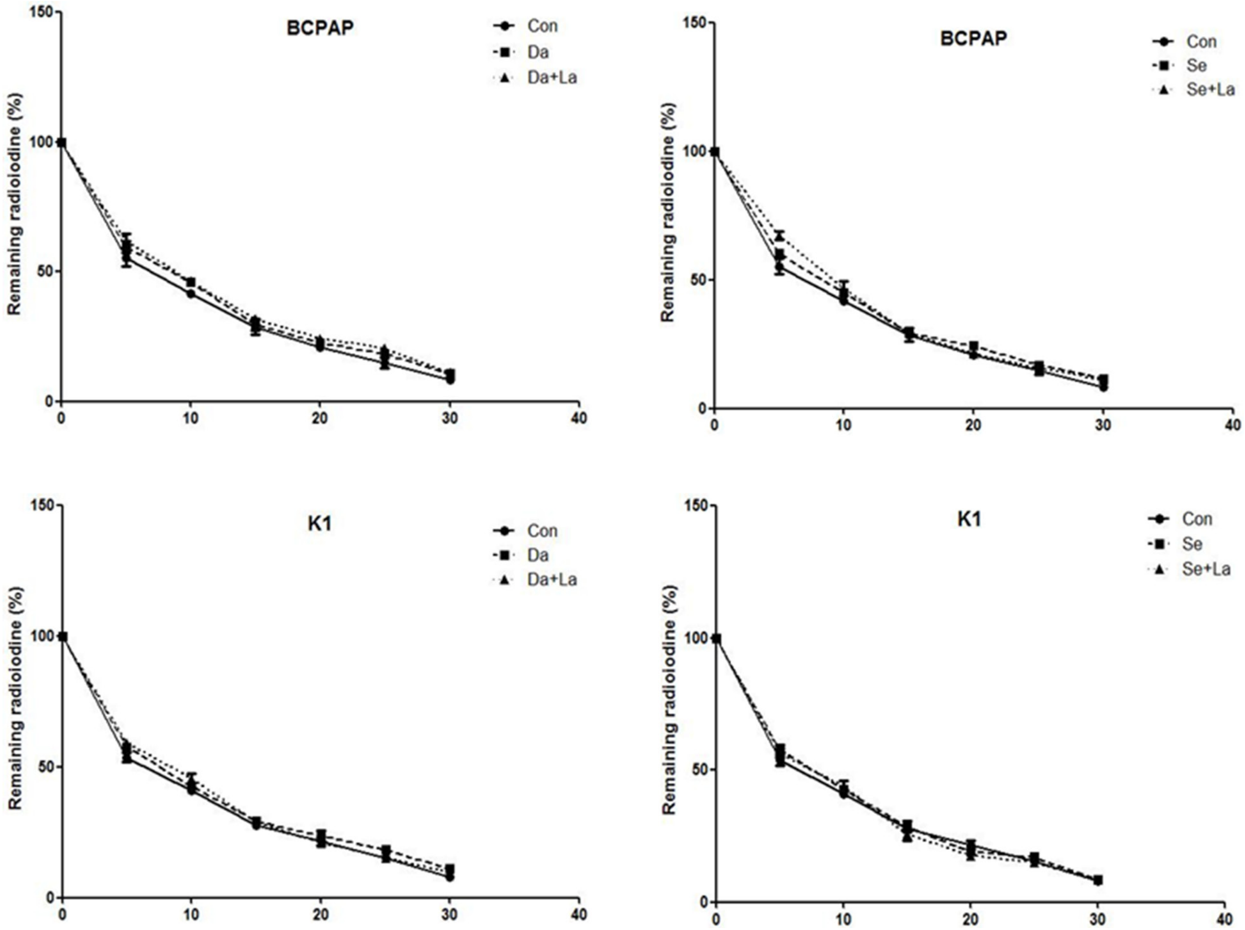

Supplementary Figure 7: Radioactive iodine efflux in BCPAP cells and K1 cells. Cells were treated with 0.1 μM dabrafenib/2.5 μM selumetinib alone or in combination with 1 μM lapatinib. Data are expressed as mean ± SD. Da: dabrafenib; Se: selumetinib; La: lapatinib.
